# Supporting breastfeeding: Tanzanian men’s knowledge and attitude towards exclusive breastfeeding

**DOI:** 10.1186/s13006-019-0244-7

**Published:** 2019-12-26

**Authors:** Janeth Bulemela, Heka Mapunda, Erna Snelgrove-Clarke, Noni MacDonald, Robert Bortolussi

**Affiliations:** 1 Tanzanian Training Centre for International Health (TTCIH), Ifakara, Tanzania; 2 St Francis Referral Hospital, Ifakara, Tanzania; 3 St. Francis University College of Health and Allied Sciences, Ifakara, Tanzania; 4 Dalhousie University, Halifax, NS Canada; 5 Queen’s University, Kingston, ON Canada

## Abstract

**Background:**

Exclusive breastfeeding (EBF) is one of the key strategies to ensure infants and young children survive and grow. However, a 2010 study showed that it was only practiced by 50% of Tanzanian women. That study also found that men were rarely supportive; either at home or in the health facilities, due to their personal beliefs or to traditional beliefs and culture of the community. In a report six years later the rate of EBF has decreased to 30%, in one region.

**Methods:**

In this qualitative study, we used focus groups to assess the knowledge and attitudes of 35 men from three villages on the benefits of EBF, the disadvantages of not breastfeeding**,** and how they can support their partners’ breastfeeding. In addition, we assessed how they felt about spending time at home, if they considered handling the infant to be rewarding and whether they helped the mother with home chores. Differences in village infrastructure and characteristics were noted.

**Results:**

Five themes were identified, including traditional roles, and feelings of exclusion/inclusion and resistance. Men felt they needed better information on EBF. They wished that their partners could breastfeed for a longer time, since they realized it improved infant growth and prevented disease; however, they did not have time to remain with the infant at home. Poverty required the men to work for long periods outside the home. As well, the men were not involved with the Reproductive Child Health Clinic (RCHC) except at the time of delivery or for mandatory HIV testing, however, they wanted to be educated together with their partners at the RCHC.

**Conclusion:**

Most men in this study understood that the EBF period was important, and that it broadened their relationship with their partner. EBF, however, could be a challenge for couples because of poverty. Nevertheless, many men wanted to help and to become more involved.

## Background

The World Health Organization (WHO) supports a comprehensive implementation plan to improve maternal and infant nutrition [1]. This plan includes six global targets for improvement by 2025. One target was to reach a rate of exclusive breastfeeding (EBF) of at least 50% at age six months [1]. In Tanzania, six years after this target was set, only 30% of mothers continue EBF for six months, even though the majority (66%) began breastfeeding their infant within the first hour of birth [2]. The Tanzanian Ministry of Health, Community Development, Gender, Elderly and Children encourages EBF, but has faced challenges with acceptance in some communities. For instance, the ‘Baby Friendly Hospital Initiative’ in Tanzania has faced challenges from limited community support [3, 4].

Knowledge about cultural practices is an important first step to understand how to bring about change in a society. In rural Tanzania, young women often leave school before secondary level education with minimal formal training in health practices. Nearly half of pregnant women deliver at home without the support of trained birth attendants [3, 4]. African cultural beliefs and practices surrounding childbirth and child feeding vary widely and can sometimes be potentially harmful [5]. In these settings, advice on feeding and infant care is usually provided exclusively by their own mothers and older women in the local community [2, 6–10].

Motivation for mothers to change practices and continue EBF is multifactorial and includes internal factors (such as knowledge and understanding by the mother) and external factors (such as cultural practice and influence of others). Among the internal factors, it has been shown that pregnant women who received counseling on optimal breastfeeding and women with more than one child were more likely to be knowledgeable on the best breastfeeding practices [11]. The importance of external factors, for example the influence of family members on maternal breastfeeding practices, has been assessed in only a few countries in Africa [12, 13]. One other external factor influencing a mother comes from her spouse or partner. Yet the role of men and their influence on EBF has not been studied.

The Tanzanian government hopes to find better strategies to promote and support healthier breastfeeding practices [2]. Traditionally, men do not discuss pregnancy or child-care with their spouse and are not involved in antenatal care [6, 14]. In this study we explored the role that men in southeastern Tanzanian villages play in EBF through an exploration of their knowledge and attitudes towards exclusive breastfeeding.

## Methods

### Design and setting

This qualitative study, using focus group discussions (FGD) with fathers, was undertaken in three villages in the Kilombero Valley, Ifakara District. Each village had one or two adjacent hamlets. This district is a large agricultural area along the Kilombero River in southeastern Tanzania. The nearest hospital is in Ifakara; the main administrative and trading centre for the region. Researchers purposefully selected three sites at varying distances from the major urban area and health facility (Ifakara). The sites had poor infrastructure with inadequate safe water, lacked electricity and citizens were subsistence farmers.

### Participants and procedures

Recruitment of men for the FGD involved several steps. First, the District Medical Officer of Health was informed of the project and his approval was obtained. He then sent a permission document to the village community leaders, the village, or area entry point. Those receiving the document were informed of the project and their respective consent was obtained. Finally, the local village leader called a village meeting where the researchers had the opportunity to explain the purpose of the study and eligibility details to the study participants. The local leaders in each of the three villages recruited men of reproductive age, who had families, until the targeted number of participants in each village (10–14) was attained. Individual informed consent was then obtained. One focus group discussion took place in each village 1–2 weeks later.

The day and time for the FGD were agreed by the majority of participants and coordinated by their respective leaders. Researchers (JB and HM) facilitated the discussion and two or three observers took field notes also noting gestures and facial expressions. All participants were free to ask questions and add points of interest. The language of the FGD was Swahili, the language widely understood and used in all three villages. Each FGD session took 1–2 h, and the sessions were voice recorded for analysis. Probing questions were asked during the FDG to explore several areas of the men’s support of breastfeeding. These areas included (a) the men’s involvement in the baby’s and family care, (b) the perceived benefit of this involvement, (c) the moral and financial support for the mother and baby during a period of EBF, (d) the men’s involvement in the decisions that followed any challenges during the EBF period, and (e) the benefits of the men’s involvement in general health care education at the local health facilities (see Table 1). The three FGD took place between 3 September and 14 September 2015.

**Table 1:** Probe Questions: Translated from Swahili with emphasis on maintaining cultural aspects

1. What benefits does the baby get with a close and loving relationship with both of his parents?	
2. Why do fathers need to be close to the baby and spend time with him/her?	
3. When is the best time, for fathers to interact with their babies?	
4. Can a baby who breastfeeds very frequently enjoy a satisfying relationship with his father?	
5. Are there any rewarding times for fathers to spend with their baby?	
6. Why does the support of a baby’s father help the mother/infant breastfeeding relationship to succeed?	
7. How common is it for the father to head off discouragement, deflect negative comments from friends and relatives, and help calm a fussy baby, and then bring the new mother food and drink while she is breastfeeding?	
8. How often does the baby’s father remind the new mother that breastfeeding is one of the most important things she can do to get their baby off to a good start in life?	
9. How often do father’s raise options of feeding when breastfeeding difficulties arise and initiate hospital consultation/expert opinion?	
10. How useful is it for the father to attend meetings or clinics with other fathers for health education on exclusive breastfeeding?	

Interviews were done in Swahili and the audio tape was translated into English by the researchers.

Qualitative analysis was done by members of the research team (JB and HM). The broad, open-ended research questions used were based on critical items identified in a literature review. Based on the responses to questions, the reviewers (JB and HM) categorized the data into themes and coded this data into either a positive or a negative response. Each of these responses were counted for the respective village of the interviewee. Themes were once again reviewed based upon the essence of the research questions and refined accordingly to accurately reflect the data. Themes were identified by consensus of the researchers analyzing the transcripts; JB, and HM.

The proposal was reviewed for scientific merit and approved for funding by Micro Research International. The Research Ethics Board of Ifakara provided ethical approval prior to our seeking agreement from the regional Tanzanian District Medical Officer.

## Results

### Village and participant characteristics

#### Participants

Thirty-five men (Village A, *n* = 10, Village B, *n* = 11 and Village C, *n* = 14) participated in the FGD. No participant withdrew from the FGD before it was completed. The participants differed in a number of ways as described in Table 2. The mean age of the participants was 29 years. Most men were married or cohabitating (91%), but 9% (*n* = 3) were widowers.

**Table 2:** Characteristics of participants in focus group discussions

Number of focus groups	3
Age	18–50 years
Marital status	
Married	25 (71%)
Widowed	3 (9%)
Co-habit	7 (20%)
Education	
Formal (at least primary school)	31 (89%)
Informal	4 (11%)

#### Themes

The themes are (a) love and responsibility (b) belonging, (c) resistance, (d) traditional roles, and (e) exclusion / inclusion.

#### Love and responsibility

This theme highlights the participants’ love for their wives and their newborn babies, while acknowledging the responsibilities they, the men, had both inside and outside of the home. Most men felt a need to communicate and to build a loving relationship with their baby. Their sharing of responsibility for the newborn also fostered love for their wife. One man described *“I will make my baby happy and healthy.”* (35 year old man, village B). Another man acknowledged that more family members, in addition to the husband and wife, bring love to the infant. He stated, *“The baby will be more cheerful than if [he/she] was with extended family (i.e. not with the mother).”* (a young man, village C).

Caring for the newborn, as expressed by a 39 year old man, enabled many of the needs of an infant to be met when both parents hold the responsibility. This man stated,” *Baby with both parents is obviously cared for (attending to) material and moral need”* (village A*).*

Some of the men either disagreed with, or had no experience of, sharing responsibility. One man was quoted as saying; *“There is no importance of fathers if mothers are around”* (45 year old man, village C). Another man, in the same village, spoke of his lack of closeness with his infant because “*most of the time the baby is with the mother so there is no closeness to it”* (a middle aged man, village C).

The love and responsibility these men felt towards their infants when their wives were breastfeeding was influenced by the time they spent with their infants and their involvement in infant care. It was unusual for most men to advise their partners on effective breastfeeding. Some men, for example, spent the whole day outside the home, only to return and make assumptions about the baby’s care. The mothers did not see infant care as a man’s responsibility and tension formed between the couple, particularly when breastfeeding was framed negatively to the mother and assumptions were made. For example, one woman was reported to have angrily shouted, “*You! Breastfeed her* (the baby) *yourself, while she knows you are a man”* (38 year old man, village A). Women were cited as saying that responsibility for infant care needs to come from the person who is in the home, not the person that is away during the day and working. A middle aged man in village A reported, *“For real. Mothers understand, it needs someone from my home or her home to come and help her to work”*. A man from another village felt that women would feel different about their responsibilities if their husbands started supporting them in the home. He said, “*The women will get used to the support and start moving to clubs and village community banks for her own good”* (a 35 year old man, village B).

Some wives in these villages accepted the support of their husbands. One young man in village C said, *“The woman will be happy and you love each other.”* Another commented, *“To spend time with [baby] brings emotion to (love for) the child and relationship strength”* (36 year old man, village B).

Many of the men, despite being aware of their responsibilities to their breastfeeding wives, did not feel that they had the time to provide such support. The time available to them to uphold these responsibilities was limited to early morning and following work. During the day, the men had work responsibilities. One middle age man stated, *“It is true the responsibility is known to us, but we do not get enough time, at least in the early morning and when you are back home, you can be asked to carry the baby in brief sessions”.*

#### Belonging

Most men agreed experiencing feelings of reward with their babies as they hugged and embraced them. Culturally, these men have a feeling of automatic ownership; the infant belongs to them. One man compared the sense of belonging as being similar to his harvest. He said, “*I feel prestige to see my wife has a baby, like as well when I harvest the crops at large. I know other men also appreciate to be called fathers of someone”* (a 39 year old man, village A). In addition, another equated that sense of belonging to the infant needing them; “*A baby will cry needing you”* (19 year old man, village C).

Some men, however, were embarrassed and dissatisfied with the situation and stated that they would move outside their home to another woman during this period of care. These men did not have a sense of belonging during this breastfeeding experience and were reluctant to discuss the matter as they saw the baby’s care as solely a woman’s responsibility. For example, a man in village B said, *“I do not think I will have frequent interactions of any kind with an infant or need to remind the mother to breastfeed, since baby is always with the mother”* (40 year old man).

#### Resistance

It was evident by the men’s conversations that they were ready and/or willing to attend to their wives’ health needs, but they did not feel they were supposed to take on this initiative. That is, they resisted this provision of support, either because of the predominant men’s culture or because the women would not permit them to do so. From the older men there was resistance to support and to discussing issues outside of the home. One man stated, *“I do not see the reason of this discussion here; it needs to be with women alone”* (50 year old man, village B).

It was evident from many of the conversions, demonstrated in the previous two themes, that resistance by men over 40 years to supporting breastfeeding is more prominent than in younger men.

#### Traditional roles

It was evident that men considered themselves as persons of wisdom and respect in rural Tanzania. Women were expected to be humble in order to gain support for breastfeeding. That is, there was a belief among men that traditionally, women respected men, talked less and did not instruct their partner. One man stated, *“She needs to respect and appreciate my personality”* (19 year old man, village C). Moreover, breastfeeding support will occur as long as traditional values are upheld.

Other men, however, saw a need to assist in roles that they considered were traditionally carried out by mothers or other relatives. Examples included *“Your relatives shall come from home to assist with the chores” (men in village A)* and *“It depends on how you were raised. Some siblings are born with only males (in some households there were only boys), thus from the beginning of the childhood they must do domestic work. And then it may be easy to do it in adulthood”* (32 year old man, village B*).* When adherence to perceived traditional roles cannot be met, as in the example above, these men were more accustomed to breaking with that tradition and supporting their breastfeeding wife.

Some men were worried when they were asked about their involvement in family care. It was their belief that home chores should be relegated to women. However, some men in our study occasionally cooked for their family, which had the potential to instigate conflict. A 34 year old man stated, *“What will be the wife’s tasks, if I cook, and likely she will get used to it and disrespect me! I do not want to be detected”* (34 year old man). If breastfeeding support came in the way of men carrying out chores, some of the men were worried that others would see that they were breaking with tradition. Some of the men even went as far as to say that *“There is a lot of work, and when you are back home, the wives have harsh language which annoys us. Some are talkative and abusive which you cannot correct”* (40 year old man, village A*).* Despite wanting to support their wives to breastfeed, perception of traditional roles was present in the FGDs. For instance, *“A woman may say: are you cooking today? This is not good for the men and it should never happen in normal circumstances”* (40 year old man, village B).

It was interesting to note that younger village men had minimal conflict when it came to involvement in pre or postnatal clinic attendance. For instance, one commented, *“Some may be the transporter of their women to the hospital, riding a bicycle and or walk together”* (18 year old man, village B*).* Adherence to traditional roles, was spoken of by most participants. The younger men, however, were more likely to not adhere to traditional roles.

#### Exclusion / inclusion

Fathers in all villages complained that they were excluded from receiving health information. Even though the younger men may have attended the clinics, the information was shared only with the women. For example, men were rarely invited to, or received, care information from the RCHCs when their partners visited during pregnancies or after delivery. Comments about the sharing of information included, *“Despite escorting your woman, you may be ignored for health education or not given any instructions. And, by nature, our women understand less how to act or what to tell after”* (31 year old man, village C). And, *“Attendance (at) clinic helps to know what is not known, otherwise the health workers used to leave the men outside the room; even though men want to learn. This will especially happen if the woman is clerked briefly, the men will not gain anything*” (34 year old man, village B).

Some men admitted to a lack of reproductive knowledge. This was seen primarily among young couples who wanted to differentiate between the old and the new generation, they wanted to be included. One man reported, “*It is good to escort the family to clinic, so that you understand early the clinical features of illnesses, and you address the problem; it brings love and courage, men then understand the information and any advice can be smoothly followed”* (40 year old man, village B).

Despite distinctions, it is clear that each theme is embedded and influenced by traditional beliefs about Tanzanian breastfeeding cultural practices.

## Discussion

The major finding from this study is that beliefs about traditional Tanzanian culture in rural villages influenced men’s attitudes towards their responsibility for family care and breastfeeding support of the mothers of their babies. Men regarded themselves as superior to their partners, which influenced and undermined support for women to EBF.

Our findings on male attitudes towards breastfeeding are similar to those of Yourkavitch in low and middle-income countries [15] and Njeri in rural Kenya [16]. Similar to our finding of ‘*exclusion / inclusion’*, there was little breastfeeding education provided to fathers in either of these studies. Both studies also noted that some men had concerns about supporting their partner and felt left out of breastfeeding. The study by Njeri noted some men felt less responsibility for infant and child care, were embarrassed when their partner breastfed in front of other people and had poor knowledge about breastfeeding [16]. When a facilitator asked these men if it was appropriate to express breast milk for the baby, all were surprised and many said they had never heard of such a possibility. Some explained that since most mothers eat poorly and work too hard, they would not produce enough milk to express and leave for the baby. One man believed that expressing milk would cause disagreement at home because it is a new practice for them [16].

Some men in our study accepted that they needed to attend to, and stay close to, their babies as this would lead to a good relationship with the baby and the mother, as identified in the theme of ‘*love and responsibility*’. Some men in our study wanted to share responsibility for their baby in order to build a better relationship with the mother. In a study conducted in Namibia, men were willing to change, even though they were not involved in decision making of infant feeding, and did not attend antenatal clinics [17]. In contrast, men in Masasi Tanzania did not wish to be actively involved with the baby’s care, perceiving their main role as being ‘breadwinners’ [14]. Still, it was recognized as important for a father to be close to his baby and spend time with the child, since the mother needs to rest before the next round of care.

The Tanzania Food Drug Authority (TFDA) published a regulation statement on EBF with guidelines to be followed and promoted by health care providers and institutions. These guidelines recommend exclusive EBF for the first six months. In our study, through the theme of ‘*exclusion/ inclusion*’, we identified that some fathers do not feel knowledgeable enough about the health of their partners and children. These men reported a need to be offered health education and to be invited to participate in the prenatal and postnatal clinics. The TFDA guidelines support working with villagers to support breastfeeding. This support may require a shift in attitudes and practices in these villages. Similar to our study, a study conducted in Masasi, Tanzania showed that men wanted to be involved in antenatal education to increase their understanding of reproductive health [14].

To prevent maternal discouragement with EBF, many men in our study, primarily the younger men of less than 22 years, deflected negative comments from friends and relatives, helped calm a fussy baby, and brought social support to the new mother while she was breastfeeding. As evidenced in our theme ‘*traditional roles*’, such support was not common with older men, which may result in inadequate breastfeeding and consequently poor health of the baby. The advice of relatives and friends is very important since men may have limited health education and less motivation to attend the prenatal clinic. In a review, a positive experience was reported when practical information on how to support their partner was provided to men [15]. This advice helped them improve EBF and as identified in this current study, provided an opportunity for men to feel included in the breastfeeding experience.

The age of the couple played a role in the encouragement of EBF in our study, as evidenced by our theme of ‘*traditional roles*’. Moreover, families and friends generally were supportive; especially when the parents were young. Older men encouraged younger men to seek help. In a study in Nigeria, only 38% of men understood the important benefits of breastfeeding and this lack of health knowledge was significantly influenced by education and age [7].

There are several limitations that should be noted in this study of Tanzanian men’s knowledge and attitudes towards breastfeeding. First, the study was conducted in Swahili. It is possible some meaning was lost in the translation to English. Second, the researchers were new to qualitative research. All efforts were made to allow the themes and the naming of the themes to emerge from the data. It was challenging due to geographical location to have all the study authors involved in the activities of analysis, however, future work might benefit from this collective approach. Third, in Tanzania community leaders or elders initially give permission to researchers to seek the consent of research participants. As was the case in this study, following permission of the community leaders, the men were invited to participate. Finally, the results of this study are centered around men of a specific area in Tanzania. It is important to note that while the results align with their respective cultural backgrounds; these results may not be representative of other men in this same area.

## Conclusions

In our study, most men understood the importance of EBF and felt pleased by their involvement in support of their partner. The men, primarily those that were older, also agreed that they needed to increase their support for their partner to EBF. Despite knowing their responsibilities at home, time spent there was limited. Poverty and hard labour away from home by the men hinders their ability to be available to support the mother’s care of their infants.

Men want to, and should be, included in pre and postnatal clinic programs on education about breastfeeding and other important sessions on reproductive health. Both parents should get education on breastfeeding together, to reinforce the importance of EBF for each parent.

## Abbreviations

EBF: exclusive breastfeeding
RCHC: Reproductive Child Health Clinic, TFDA, Tanzanian Food and Drug Authority
